# Gut Function-Enhancing Properties and Metabolic Effects of Dietary Indigestible Sugars in Rodents and Rabbits

**DOI:** 10.3390/nu7105397

**Published:** 2015-09-28

**Authors:** Jin Xiao, Barbara U. Metzler-Zebeli, Qendrim Zebeli

**Affiliations:** 1 Department for Farm Animals and Veterinary Public Health, Institute of Animal Nutrition and Functional Plant Compounds, University of Veterinary Medicine Vienna, Vienna 1210, Austria; jin.xiao@vetmeduni.ac.at; 2 University Clinic for Swine, Department for Farm Animals and Veterinary Public Health, University of Veterinary Medicine Vienna, Vienna 1210, Austria; barbara.metzler@vetmeduni.ac.at; 3 Department for Farm Animals and Veterinary Public Health, Institute of Animal Nutrition and Functional Plant Compounds, University of Veterinary Medicine Vienna, Vienna 1210, Austria

**Keywords:** indigestible sugars, cecal fermentation, rodent, rabbits, mineral absorption

## Abstract

Indigestible sugars (iS) have received particular interest in food and nutrition research due to their prebiotic properties and other health benefits in humans and animals. The main aim of this review article is to summarize the current knowledge regarding digestive and health-enhancing properties of iS such as sugar alcohols, oligosacharides, and polysaccharides, in rodents and rabbits. Besides ameliorating gut health, iS ingestion also elicits laxative effects and stimulate intestinal permeability and fluid secretions, thereby shortening digesta transit time and increasing stool mass and quality. In rodents and rabbits, as hindgut fermenters, consumption of iS leads to an improved nutrient digestibility, too. Cecal fermentation of iS reduces luminal pH and extends wall tissue facilitating absorption of key dietary minerals across hindgut. The microbial fermentation of iS also enhances excessive blood nitrogen (N) flowing into the cecum to be used as N source for bacterial growth, enhancing N retention in cecotrophic animals. This review also highlights the impact of iS on improving lipid metabolism, mainly by lowering cholesterol and triglycerides levels in the blood. The paper serves as an index of the current knowledge of iS effects in rodents and rabbits and also identifies gaps of knowledge that need to be addressed by future research.

## 1. Introduction

Indigestible sugars (iS) include a large assembly of simple and complex carbohydrates including monosaccharides, disaccharides, oligosaccharides, and most sugar alcohols which have in common their crystalline nature, solubility in water, and, most importantly, resistance to host enzyme hydrolytic activity in the small intestine in humans and monogastric animals [1,2,3,4]. Due to their indigestibility, iS pass through the upper gastrointestinal tract and become readily available for microbes in the hindgut where they are degraded through various metabolic pathways to short-chain fatty acids (SCFAs) and gases. These degradation processes occur in the lumen and on the interface between the lumen and the gut epithelium [5]. In being water-soluble, microbial degradation metabolites can pass the intestinal barrier either through active absorption processes or facilitated diffusion. Both the chemical structure of iS and the glycosidic linkages between their molecules have a great impact on the site where the different iS are fermented and how the iS influence the physiology of the gastrointestinal tract [2]. In addition, due to the indigestibility and slow active transport across the small intestinal mucosa iS can increase the amount of osmotic active molecules in the intestinal lumen which, in turn, lead to an increased luminal water influx and thus contribute to the laxative properties of iS [3].

The rising popularity of so-called bioactive iS including, among others, disaccharide difructose anhydride (DFA) III and various oligosaccharides, such as fructooligosaccharides (FOS), xylooligosaccharides (XOS), mannanoligosaccharides (MOS), and galactooligosaccharides (GOS), have accelerated food science research in this area over the past two decades. Apart from their implication as low-calorie sweeteners and humectants, indigestible polyols (sugar alcohols), which are chemically defined as saccharide derivatives in which a ketone or aldehyde group is replaced by a hydroxyl group [6], such as maltitol, mannitol, sorbitol, erythritol and xylitol, have also been evaluated for their gastrointestinal health enhancing properties. Owing to their promising physiological effects, the interest in fortificating diets with iS has greatly increased and a large body of evidence in humans and animals has documented beneficial effects of using the various iS to ameliorate gut function and health as well as nutrient digestion and metabolic health [7,8,9].

The most extensively used animal models to examine physiological effects of iS include rats, mice, hamster, guinea pigs, and rabbits [10,11,12,13,14], and to a lesser extent the pig [15]. Rabbits, guinea-pigs and hamsters are small herbivores, whereas rats, mice and pigs are omnivores. Compared with large herbivores, such as ruminants and horses, these small herbivores have a lesser ability to break down and utilize dietary fiber, but they require more energy and protein per unit body mass than larger herbivores [16]. As hindgut fermenters, rodents and rabbits comprise either a large cecum or colon, where digesta is retained, mixed and fermented by microbes [16]. Rabbits have a large and sacculated cecum, which contain 40% of gastrointestinal digesta [17]. However, compared to rabbits, guinea pigs possess a larger cecum, containing 65% of gastrointestinal digesta and longer colon [17]. The colon in guinea pigs is capacious and never empty, which have a stronger ability to digest dietary fiber [18]. The hindgut of hamsters is lighter and shorter than that of rabbits and guinea pigs, but it is larger than in rats [19]. Chiou *et al.* [19] and Yu *et al.* [20] compared digestive function among rabbits, guinea pigs, rats and hamsters (Table 1). Guinea pigs showed highest crude fiber digestibility due likely to their greatest relative weight and longest length of colon-rectum, compared with other animals. Fiber-hydrolytic activity in the cecum of guinea pig was highest, too [20]. In contrast, rats showed the lowest fiber hydrolytic activity in the cecum and smallest relative weight of the hindgut, which corresponded to the lowest capability of the latter animals to digest fiber (Table 1). Rabbits possess the longest retention time of digesta in the gastrointestinal tract, followed by guinea pigs, hamsters and rats, which was the shortest [19]. The passage rate of digesta through the hindgut seems to be related to the relative length and the degree of sacculation of the local section [21]. In addition, the short dense and folded mucosa appears primarily in rabbits, and followed by guinea pigs [19]. Thus, compared with rats, hamsters and mice, rabbits and guinea pigs may provide larger volume for iS fermentation and production and absorption of SCFA. The total bacteria concentration and pH in the hindgut in rabbits, guinea pigs, rats and mice are similar [21]. However, the species, population and distribution of bacteria in rabbits are closer to the colonic microflora in humans [21]. Indeed, similarities and major differences in the gut physiology and microbiota between rodent models and humans have been recently reviewed [22,23]. When rodents and rabbits are used as animal models to study the effects of iS in human gut, metabolic limitations exist due to the anatomical, physiological and microbial differences between these animals and humans [21,22,23]. For example, differing from rodents and rabbits, the cecum is a small dead-end pouch forming, and has less bacterial activity in humans [21]. In contrast, humans have longer and more sacculated colon which consists of four sections called the ascending colon, the transverse colon, the descending colon and sigmoid colon [22]. Human colon contains larger population of bacteria in the gastrointestinal tract than the rodents and rabbits do [21,22]. Therefore, after the ingestion of iS, the colon is responsible for iS fermentation in humans. The colon also is the major site of production and absorption of SCFA, in addition, absorption of water, salts and minerals in humans.

**Table 1:** Comparison of weight and length of hindgut, fiber digestibility and total SCFA concentration in rodents and rabbits.

Items	Rabbits	Guinea Pigs	Rats	Hamsters
Relative Weight of Gastrointestinal Segment (g/100 g Body Weight)				
Large intestine	9.9 ^b^	14.1 ^a^	4.4 ^d^	6.0 ^c^
Cecum	7.8 ^b^	9.2 ^a^	2.4 ^c^	3.2 ^c^
Colon-rectum	2.1 ^c^	4.9 ^a^	2.0 ^c^	2.8 ^b^
**Length of Gastrointestinal Segment (cm)**				
Cecum	63.8 ^a^	11.3 ^b^	4.9 ^d^	7.3 ^c^
Colon-rectum	98.5 ^a^	86.8 ^b^	18.8 ^d^	34.5 ^c^
**Apparent Digestibility of Nutrients (%)**				
Crude fiber	21.1 ^b^	51.3 ^a^	7.4 ^c^	25.5 ^b^
NDF	30.0 ^c^	55.0 ^a^	26.1 ^d^	38.4 ^b^
ADF	23.4 ^b^	51.7 ^a^	11.2 ^c^	25.1 ^b^
**Total SCFA Concentration (umol/g)**				
Cecum	73.0 ^b^	40.9 ^c^	179.4 ^a^	85.0 ^b^
Colon-rectum	64.4 ^b^	35.2 ^c^	149.2 ^a^	60.3 ^b^

Source: Chiou *et al.* [19] and Yu *et al.* [20]. Data are shown as mean values; ^a–d^ means within the dame row without the same superscripts are significantly different (*p <* 0.05); SCFA: short chain fatty acids; NDF: neutral detergent fiber; ADF: acid detergent fiber.

In this article, we summarize beneficial effects of non-digestible monosaccharides, disaccharides, oligosaccharides and sugar alcohols including prebiotic effects and the related desirable physiological changes in the large intestine after oral ingestion of various types of iS as well as their putative positive health benefits on nitrogen, mineral and lipid metabolism using results originating mainly from rodents and rabbit research. Despite differences mentioned above, rodents are often used as gut and metabolic models in humans; therefore, a few examples of beneficial effects of iS in this article also come from human studies.

## 2. Prebiotic Properties of iS and Effects of Fermentation

**Figure 1:**
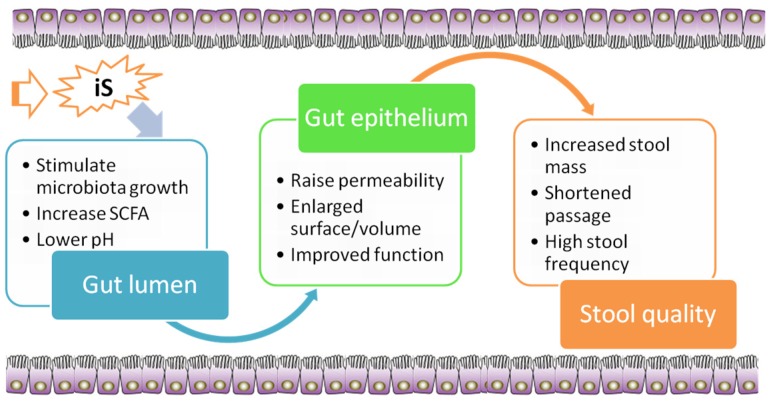
Summary of prebiotic effects of dietary indigestible sugars (iS) in the large intestine.

Well-known nutrition-based strategies to modulate the gut microbiota and sustain gut homeostasis are based on the use of dietary fiber and analogous carbohydrates [24]. Due to many benefits of iS in the prevention and control of chronic human diseases and the fact that many of these effects are mediated via stimulation of fermentation in the large intestine, there is ongoing research interest in evaluating the potential of iS to be used in diet preparations as prebiotic [25]. A prebiotic is generally defined as a selectively fermented dietary ingredient that confers a health benefit on the host in association with modulation of the intestinal microbiota or microbial activity [26]. Various known prebiotic effects of iS in rodent and rabbit models are discussed in this section and are summarized in Figure 1.

### 2.1. Selective Bacterial Proliferation and Fermentation

The main end products of the fermentation of iS are SCFAs, predominantly acetic acid, propionic acid and butyric acid, and lactic acid, as well as water, various gases (carbon dioxide, hydrogen, methane), bacterial cell biomass [27,28] and heat. The production of SCFAs is influenced by several factors, including the number and microbes present in the large intestine [26], type and mass of substrate [29], and gut transit time [30]. Thus, different types of iS lead to diverging SCFAs patterns in the hindgut. For instance, FOS increased mainly butyric acid and acetic acid concentrations in the human colon [31]. Mannitol, in turn, has showed to stimulate the generation of lactic acid, succinic acid and butyric acid and to reduce that of acetic acid in the hindgut and feces of rats, whereas inconsistent reports exists regarding propionate fermentation [32,33]. Difructose anhydride III feeding to rats increased especially acetic acid formation [34] which could be linked to the enhanced abundance of *Ruminococcus sp*. M-1 withdietary DFA III.

Traditionally, the main health benefits of iS consumption on the intestinal microbiota have been related to selective promotion of saccharolytic bifidobacteria and lactobacilli species, while harmful bacteria such as *Enterobacteriaceae*, *Clostridium sensu strictu*, *Streptococcus faecalis* and *Proteus* sp. typically decreased following iS intake in human large intestine [35,36]. For instance, FOS intake induced a significant increase in the population of *Lactobacillus* spp. and *Bifidobaterium* spp., which came along with increased levels of butyric acid and acetic acid in human colon *in vitro* [31]. Of particular interest is the promotion of butyric acid-producing bacteria (e.g., *Faecalibacterium prausnitzii* and *Eubacterium rectale/Roseburia* spp.) through iS due to the beneficial effects of butyric acid on colonic health, as reported in humans [37] and rats [38]. Moreover, iS effect on microbiota in the different intestinal segments differs, which can be related to progressing fermentation of iS. In this regard, FOS increased fecal gene copy number of *Bifidobacterium* spp., and mediated a decrease in gene copies of total bacteria, *Bacteroides-Prevotella-Porphyromonas* group, and *Clostridium* clusters XI and XIVa, *Enterobacteriaceae* and *Clostridium difficile* toxin B in feces of rats [38]. In the cecum, in turn, FOS increased *Bifidobacterium* spp. and *Clostridium* cluster XI [38].

Apart from the direct effect of substrate preferences of bacteria and competition for carbohydrates among bacteria, the drop in large intestinal pH due to enhanced fermentation of iS inhibits the growth of acid-sensitive bacteria including some intestinal bacterial pathogens such as *Enterobacteriacae* [37], *Salmonella* [39] and *Clostridium* species [40,41], *Escherichia coli* [42] and provides a growth-advantage of more acid-resistant bacteria, including lactobacilli and bifidobacteria that withstand lower environmental pH [43,44,45,46].

Moreover, *Lactobacillus* and *Bifidobacterium* species are well recognized to suppress opportunistic intestinal pathogens such as *Bacteroides*, *Salmonella enteric*, *Clostridium diffile*, *Staphylococcus*, and *Enterobacter* in gastrointestinal tract via production of antimicrobial metabolites [44,47,48]. Stimulation of lactobacilli and bifidobacteria species by iS may therefore contribute to normal gut function and maintaining host health in a number of ways. GOS supplementation at 2% (*w*/*v*) was the optimum dosage for growth of *Lactobacillus acidophilus* NCDC13 after 12 h culture *in vitro* [49]. In rats, dietary GOS was observed to increase lactobacilli counts and decrease coliform counts in feces [50]. In addition, dietary DFA III decreased the population of bacteria related to *Bacteroides acidofaciens* and uncultured bacteria within the *Clostridium lituseburense* group and increased the population of bacteria related to *Bacteroides* *vulgatus*, *Bacteroides uniformis* and *Ruminococcusproductus* in the rat cecum [34].

### 2.2. Effects of Microbial Metabolites on the Gut

Short-chain fatty acids are an important nutrient source of the gut epithelium [51]. Especially butyrate stimulates cell proliferation of the cecal and colonic mucosa in humans and animals like pigs and rats [52,53,54,55]. After first pass uptake, the remaining butyrate and propionate are metabolized by hepatocytes. In addition, 50%–70% of acetate is taken up by the liver, thereby contributing to energy intake of the host. Roberfroid [26] reported that muscle cells generate energy from the oxidation of residual acetic acid. Increased luminal generation of SCFAs in the large intestine has been related to epithelial cell proliferation after the ingestion of iS [56]. For instance, dietary MOS supplementation has been shown to enhance cecal SCFAs concentrations in rabbits which was associated with longer intestinal villi and an increased intestinal absorptive surface [57,58].

Emerging evidences from human researches also suggest positive effects of certain SCFAs on gut mucosal immunity and attenuation of the inflammation in the large intestine. For example, butyrate can be protective against carcinogenesis [59], or act anti-inflammatory in colon cancer cells [60]. In addition, newer research has indicated that butyrate regulates gut mucosal homeostasis and elicits modulatory effects on dendritic cells [61] and on the differentiation of colonic regulatory T cell [62,63,64]. A new study in rats also showed that a short term (1 week and 2 weeks) ingestion of FOS significantly increased IgA and mucin concentrations in cecum, but this effect was attenuated with a prolonged ingestion [65], indicating development of a state of tolerance due to long exposure of this dietary treatment. Indeed, most of the studies dealing with immune-stimulating effects of iS ingestion are done in short-term studies. More long term designed studies are warranted to clarify the role of iS ingestion in modulation of mucosal immunity.

### 2.3. Enlargement of Large Bowel Size

**Figure 2:**
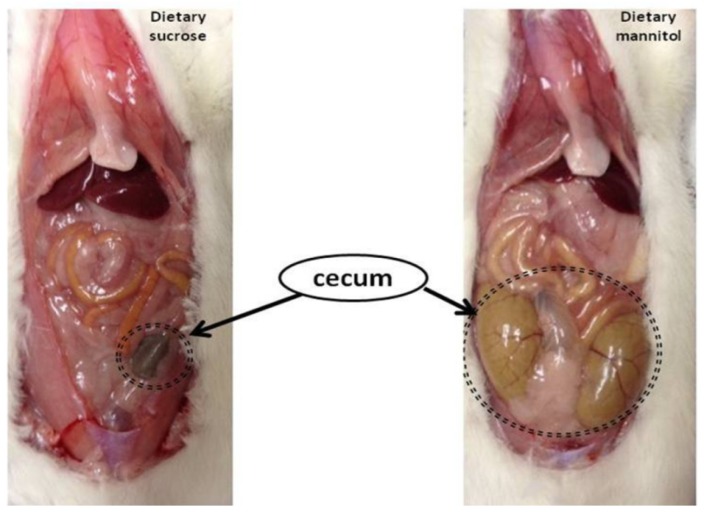
Differences in the size of cecum in rats fed sucrose or mannitol in the diet as digestible and indigestible sugars, respectively [70].

An enlargement of intestinal bowel has also been recognized as one beneficial physiological effect of iS ingestion. Important implications of enlargement in large bowel size are greater fermentation capacity and an increasing role of the hindgut in nutrient absorption in those animals. In rats, greater substrate availability for fermentation in the large intestine, due to mannitol supplementation, led to an enlargement of cecum compared to rats consuming digestible sugars such as sucrose (Figure 2). Indeed, in this research, as shown in Figure 2, dietary mannitol supplementation resulted in multifold larger and heavier cecum than in rats fed dietary sucrose.

Bigger hindgut in response to iS feeding is a typical finding in rodent studies [9,12,66,67,68,69]. For example, research conducted by Pan *et al.* [68] revealed that daily gavage of FOS, GOS, MOS, and chitooligosaccharides (delivered in a quantity of 1 g/kg body weight) to the mice for two weeks led a significant increase in the total weights of cecum and colon, associated with a reduced cecal pH. The cecal SCFAs and total anaerobes in the cecum were increased by these 4 oligosaccharide supplementations as well. When FOS was fed to rats, an enlargement of cecum and a decrease in cecal pH was reported, too [69]. In hamsters, 16% inulin feeding increased total cecal weight and cecal wall weight as well [12].

### 2.4. Change in Intestinal Transit Time and Increase Stool Mass

Indigestible sugar supplementation in the diet has been shown to increase stool mass and shorten transit time of digesta through gastrointestinal tract. For example, the gut transit time was significantly reduced with 6% FOS supplemetation in rats [66]. The increased stool mass has mainly been associated with higher amounts fecal water and greater amounts of bacterial biomass [35]. In addition, the fermentation of iS is believed to result in greater production of microbial biomass in large intestine, increasing this biomass in feces. Indeed, the bacterial biomass accounts for at least 30% on dry matter of feces [35], which is responsible for the increased fecal bulk and dry matter excretion. Daily ingestion of GOS (3.5 and 7 g/day) enhanced fecal biomass of *Bifidobacteria* in patients suffering from irritable bowel syndrome [71].

The role of iS consumption on stool weight and size has also be related to modulation of the transit time by iS fermentation in the large intestine. Particularly butyric acid seems to impact the transit time through the gastrointestinal tract, as greater proportions of butyric acid lead to a shorter cecal transit time [30]. Oral ingestion of FOS effectively increased stool frequency, shortened the transit time of digesta and increased fecal weight in mice [72]. In human studies, the ingestion of FOS significantly increased the bowel evacuation rate, reduced perception of straining effort and pain, and improved the quality of stools [72]. A specific mixture of GOS and long-chain FOS enhanced stool frequency, led to softer stools [73], and accelerated gastrointestinal transport in infants, too [74]. In addition, XOS intake increased the population of intestinal *Bifidobacterium* in humans, maintained normal fecal water content, and reduced constipation [75]. Therefore, iS could be considered as useful and safe tool for ameliorating constipation.

Indigestible sugars alter the intestinal permeability in two ways: (1) through their physical presence, and (2) through fermentation. Physical presence affects several physiological functions in the upper intestine. Indigestible sugars produce high osmotic pressure, accumulate fluid within the lumen to maintain isotonicity, and increase the permeability of the intercellular junctions in small intestine in rats [76]. On the other hand, excessive consumption of iS may induce osmotic diarrhea [77]. In large intestine, osmotic pressure is caused by the high production of SCFAs during the fermentation of iS. The transport process of SCFAs is accompanied by increased Na, K, and water absorption [78]. An increased fluid secretion through a greater luminal osmotic pressure results in softer stool. Therefore, osmotic effect of iS can be considered as one of the reasons for their laxative effects besides increasing the bacterial biomass.

## 3. Effects on Nutrient Digestion, Absorption, and Systemic Metabolism

### 3.1. Enhancement of Mineral Absorption

**Figure 3:**
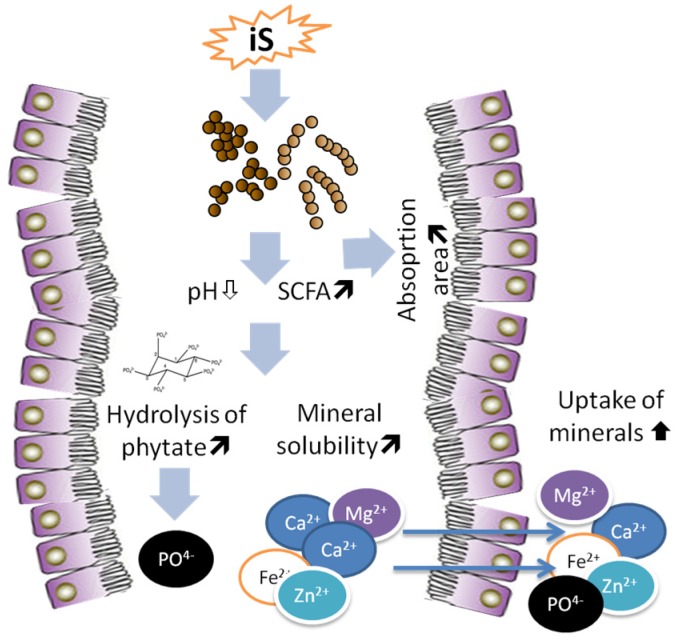
Description of the effects of dietary indigestible sugars (iS) on formation of short chain fatty acids (SCFA) and mineral absorption in the hindgut in rats.

Improvement of mineral bioavailability is one important properties of iS, which when fermented in large intestine stimulate microbial activity, SCFAs formation, and eventually an enlargement of cecum, thus playing an important role in mineral absorption (Figure 3). Indeed, increasing evidences suggest that iS supplementation promoted mineral absorption including Ca, P, Mg, Zn, and Fe with key physiological functions in the body. Most of the scientific evidences of the effects of iS is based on the results of experiments with rats, whereby iS increased the availability of Ca, Mg, Fe and Zn [71,79,80,81,82,83], which are the most important mineral elements for bone mineralization and bone health [84]. The ingestion of lactosucrose, which is synthesized from sucrose and lactose, increased the apparent Ca absorption and Ca accumulation in the bone of rats [85]. Supplementation of FOS in the diet enhanced the mineral absorption, and, most importantly, counteracted the adverse effects of phytic acid in mice [69]. This is highly important because dietary P is mostly in form of phytic acid in cereal- and legume-rich diets, which is largely unavailibale for the host animal [86]. Thus, conteraction of phytic acid by iS supplementation [56] increases the availability of dietary P [86].

Other research also found that dietary GOS enhanced net absorption of Ca and Mg, as well as their retention, and femur and tibia breaking strength in rats [87]. Both the distal femur, trabecular volumetric bone mineral density, and proximal tibia volumetric bone mineral density increased with GOS supplementation, too [87]. Feeding of GOS for 9 days to growing rats with hypochlorhydria significantly improved apparent absorption of Ca, Mg and Fe [82,88]. In particular, the effect was stronger when GOS ingestion was combined with a dairy product fermented by *Lactobacilli*, resulting in improved Ca, P, Fe and Zn retention during the mineral balance test in rats [88]. Ingestion of FOS has also been proven to improve intestinal absorption of Ca, Mg, Fe, Zn, and Cu in rats [54,89,90,91]. Ingestion of indigestible polyols increased Ca content in bone [92,93,94,95] and bone strength [96,97] in rats. Dietary mannitol has been shown to increase Ca and Mg absorption and their retention in the bone of rats, too [33].

Typically, beneficial effects of iS on enhancing the bioavailability of minerals were combined with improvements of SCFAs formation, decreased luminal pH, and enlargement of intestinal wall tissue in the above researches. Hydrolysis of phytic acid contained in the cereal-based diets via iS fermentation [69] may also contribute to a higher bioavilibility of P and other minerals such as Ca, Mg, Zn, which normally are bound by phytic acid [86,89].

Following the ingestion iS, the absorption of minerals is shifted towards the large intestine in rats [98]. Dietary DFA III increased Fe absorption through expansion of cecal mucosa [99]. Cecum is the main segment with highest Ca absorption in the rat intestine, with active transport of Ca in cecum being several times faster than in duodenum, proximal colon and distal colon in rats [100]. Under normal conditions, cecal epithelium absorbs free-ionized minerals released from insoluble mineral complexes in the presence of small acidic molecules, such as acetic, propionic, butyric, succinic and lactic acids which are formed during the fermentation of iS by luminal microbes [98,101]. Indigestible sugars fully or partly reach the cecum and are fermented there, producing SCFAs which are responsible for the drop of cecal pH in rats (Figure 3). It was shown that inulin and FOS improved mineral absorption and the effects were associated with SCFAs formation and reduced luminal pH [102]. The drop of cecal pH increases the concentration of mineral cations [103,104]. Raschka and Daniel [105] reported that a lower pH increased the amount of soluble and ionized minerals, which was adequate for their absorption. 

Enlargement of cecum and colon induced by iS consumption and the resulting SCFAs provide extensive absorption area to stimulate mineral absorption in trials involving piglets, rats, and chicken [33,52,69,106,107]. Subsequently, the SCFAs may directly affect intestinal mineral absorption by stimulating the intestinal epithelium and increasing its absorptive capacity and by increasing intestinal blood flow and fluid and electrolyte uptake, as reported in human colon [108]. Short chain fatty acids, especially butyrate formed by iS fermentation, increase the energy supply to intestinal epithelial cells and regulate the electrolyte exchange of minerals and hydrogen to facilitate mineral absorption by the epithelial cells in the rat colon [109].

Moreover, iS ingestion increases bone mineral accumulation, where by this effect depends on the dose and type of these sugars and mineral level in diet of rats [110]. The benefit function is most effective when high levels of minerals in the diet are supplemented in rats [101]. Feeding Fe-deficient rats with GOS together with FeCl_3_ increased hemoglobin (Hb) synthesis (by 17%) and mean Hb concentration in the erythrocytes relative to FeCl_3_ level in the diet [13]. Dietary heme iron did not prevent postgastretomy anemia alone, but dietary FOS increased iron absorption and prevented anemia when its consumption was combined with iron citrate or heme iron in gastrectomized rats [111].

In these years, the major part of researches on iS has been designed in mineral deficient animals or animals with mineral malabsorption. Ohta and colleagues confirmed that dietary FOS enhanced Mg absorption and the absorption of Ca, Mg and Fe in Mg-deficient rats and Fe-deficient anemic rats respectively [112,113]. As well, dietary supplements of GOS, FOS, DFA III, polydextrose and epilactose were reported to increase Ca and Fe bioavailability and prevent anemia in gastrectomized animal models [111,114,115,116,117].

Ovariectomy has been well known to induce bone mineral loss and osteopenia. Mineral absorption such as Ca, Mg and Fe was increased and bone mineral loss was prevented by dietary FOS, GOS, DFA III, maltitol and polydextrose in ovariectomized rats [106,110,118,119]. Pieces of evidence suggest that Ca retention was greater after 11 day supplementation of iS, but no longer after 25 days in rats [120]. Therefore, the effects of iS on mineral absorption and retention may be related to the length of feeding period too. Because most of the studies are conducted during short periods of time, elucidation of this aspect requires further research with long-term designed studies.

An increasing body of evidence suggests that iS enhances mineral absorption across small intestine wall, too. For instance, dietary lactose induced stimulation of Mg absorption in rats, and this was caused by a lowering effect on ileal pH [121]. Using a tracer technique with ^45^Ca, maltitol was found to stimulate Ca absorption in rats, and osmotic activity of maltitol in small intestine was suggested to contribute to the increase in Ca absorption [122]. Other iS such as melibiose, DFA III, or DFA IV increased the permeability of intercellular passage, affected the epithelial tissue and opened the tight junctions of jejunum, ileum, cecum, and colon of rats [123]. Absorption of Ca, Mg, and Zn via the paracellular route was enhanced, thereby promoting Ca, Mg, and Zn absorption in the small intestine and large intestine *in vitro* [123,124]. In these *in vitro* studies, the stimulating effect of iS on mineral absorption was related to greater epithelial permeability, opening the tight junctions to facilitate mineral absorption in small intestine.

In a small number of studies, the researchers found that iS supplementation also have similar promoting effects on mineral bioavailability in rabbits and other rodents except of rats. Dietary mannitol increased ash digestibility in adult rabbits [125,126]. A research by Kawasaki *et al.* [10] showed that dietary FOS resulted in significantly higher ash digestibility in guinea pigs when compared with dietary glucose.

### 3.2. Nitrogen Utilization

Fermentation of iS in the hindgut serves as the source of energy and carbons for bacterial growth leading to a distinguished rise of bacterial biomass. Besides available energy, dietary N is also needed for bacterial proliferation, whereby bacteria use ammonia N for their protein synthesis. Thus, the higher the availability of energy and fermentation substrates (*i.e.*, iS sources), the greater is the need of the hindgut bacteria for ammonia N, needed for their rapid growth. In rats, supplementation of dietary mannitol at 8% lowered the digestibility of crude protein compared with dietary sucrose [127]. In addition, in rats, feeding diets containing GOS or FOS at 100 g/kg decreased the apparent digestibility of crude protein when compared with the diet including the same dose of sucrose [128]. During iS supplementation period, the fermentation of these substrates resulted in a greater flux of urea N transfer from blood into cecum to provide N for cecal bacterial proliferation. Dietary FOS and XOS increased N cecal flux from blood urea N into cecum too, associated with greater cecal N excreted in feces as bacterial N [129]. This circulation of N can explain the decreased crude protein digestibility in rats when they fed dietary sources of iS. Therefore, more urea N is excreted in feces when bacterial mass increased due to iS fermentation, coupled with a reduction in urinary N excretion and plasma urea concentration. For example, when FOS and XOS were fed at 7.5 g/100 g in rats, blood urea and urinary N were reduced by 20%–30% [129].

The greater amount of bacterial biomass in fecal dry matter plays an important role in cecotrophic animals like rabbits and guinea pigs. Because rabbits and guinea pigs are typical cecum fermenters, and have a special reingestion system called cecotrophy which happens less in rats. Rats also eat their feces, but this action only happens when rats are under the condition of starving. Cecotrophy allows rabbits and guinea pigs to reingest their microbial product in the cecum, called cecotrophs or soft feces. Fecal N source includes the part of dietary protein escaped from digestion in the small intestine, endogenous proteins secreted from pancreas and intestine, mucosa cells sloughed from intestinal wall, blood urea that diffuses across the intestine with water movement, and intestinal bacteria. Bacterial protein is the major part of cecotrophs N [130]. Cecotrophs is formed in the cecum without any major differences in their composition when it is excreted [131]. Li *et al.* [125] revealed that crude protein concentration in cecotrophs was raised by iS feeding when rabbits were prevented from cecotrophy, and N excretion in urine was simultaneously reduced. The raised N in cecotrophs results from bacterial proliferation which is stimulated by iS in the cecum. The fermentation of iS induces excessive amounts of blood urea flowing into the cecum [132], which is transformed to ammonia by bacterial ureolytic activity [133] to contribute to bacterial N synthesis [128,134]. As a result, urinary excretion of N is shifted to fecal excretion [10,128]. The cecotrophs with a rich bacterial N are eaten by rabbits and guinea pigs, and bacterial N is absorbed in small intestine to increase N retention [135]. Rabbits fed 1 g/kg dietary MOS had higher overall final live weight, weight gain among the diets containing several probiotics and prebiotics [14]. In asimilar research in guinea pig, it was shown that 5% dietary FOS depressed urinary N excretion, and increased N retention [10]. The increased N retention caused by dietary iS supplementation in rabbits and guinea pigs were attributed to cecotrophy in these animals [10,14].

**Figure 4:**
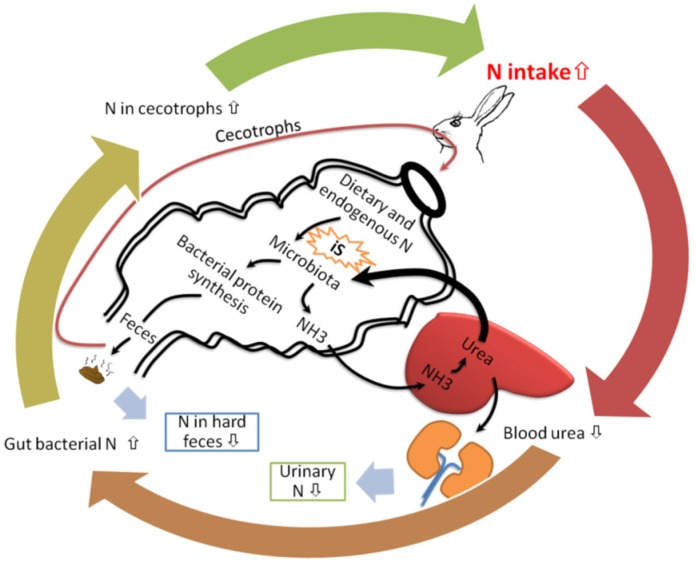
Effect of dietary indigestible sugars (iS) on nitrogen (N) metabolism and utilization in rabbits and guinea pigs.

The improved utilization of dietary N in rabbits and guinea pigs by iS intake is summarized in Figure 4. Nitrogen from the diet in rabbits and guinea pig is metabolized in a circle, a physiological process called N recycling [134]. Indigestible sugars stimulate blood urea move to cecum to be used as N source for bacterial proliferation, raising bacterial protein in soft feces [136]. Soft feces are reingested through cecotrophy. Cecotrophy has considerable nutritional significance for N supplementation in rabbits. It can provide up to 30% of daily N intake, which is mostly derived from cecal microbes. The utilization of dietary urea is more effective to increase N retention in rabbits when iS are supplemented in the diet [136]. Therefore, the positive effects of iS in cecotrophs N can make a significant contribution to meeting the N requirements in cecotrophic animals (Figure 4).

### 3.3. Role in Improving Blood Glucose and Insulin Secretion

Indigestible sugars are not directly converted into energy because of the resistance to be digested and utilized in the small intestine. Therefore, they do not stimulate an increase in blood glucose and insulin secretion. Oral administration of FOS did not change the level of glucose, fructose, and insulin in plasma, indicating that FOS was not absorbed directly into blood both in rats and humans [137]. In addition, the consumption of FOS did not result in changes of serum lipid profiles and glucose levels, but led to 3.6 fold less serum insulin concentrations compared to sucrose diet in rats [138]. FOS in the diet caused a decrease in serum glucose after 14 days addition in subjects with diabetes type II, but this effect was not shown when FOS was supplied in healthy subjects [139]. Blood glucose, total serum cholesterol and liver ascorbic acid were reduced by the ingestion of galactitol, mannitol, as well as xylitol in rats, too [140]. Sangwan *et al.* [50] found that dietary GOS decreased diabetes-associated markers including fasting blood glucose, haemoglobin, glycosylated haemoglobin triglycerides, total cholesterol, low density lipoproteins, creatinine, and urea in alloxan-induced diabetic rats. In human research, matitol and short chain FOS containing food resulted lower blood glycaemic and insulinaemic responses than the food with traditional sugars [141]. Plasma glucagon level was decreased by dietary sorbitol and dietary xylitol, but plasma insulin and glucose was little affected by the addition of these iS sources in rat studies [142].

### 3.4. Lipid Metabolism

The ingestion of iS decreased feed efficiency and body weight gain in rats, which has been mainly attributed to a decreased fat digestibility [127]. The latter research showed that dietary mannitol decreased the level of serum triglycerides and fat accumulation in body in a dose-response manner [127]. A similar result was shown with dietary sorbitol and xylitol which decreased plasma triglyceride and cholesterol, and fat tissue weights in rats [142]. Matitol supplementation suppressed weight gain, hepatic fat degeneration, hyperglycemia and hypercholesterolemia in mice fed high-fat diets [143] In addition, the epilactose in the diet lowered the levels of plasma total cholesterol and low-density lipoprotein cholesterol in rats [144]. Dietary FOS also showed a hypolipidemic effect in rats, for example, decreasing total cholesterol and triglycerides in blood [145]. In addition, a 12-week intragastrical administration of FOS reduced body weight, blood cholesterol and triglycerides in rats with fructose-induced obesity [146]. Dietary inulin lowered plasma cholesterol and triacylglycerol in hamsters [12], and the related mechanisms were postulated that inulin ingestion altered hepatic triacylglycerol synthesis and very-low-density lipoprotein (VLDL) secretion and impaired rebsorption of circulating bile acids. Bile acids excretion in feces was increased by inulin ingestion [12]. Cholesterol is used to synthesize new bile acids in the liver to compensate the loss of bile acids, resulting in lowered levels of serum cholesterol [147]. Taylor and Williams [148] demonstrated that the consumption of FOS resulted in marked reductions in triglycerides, and to a lesser extent cholesterol levels, because FOS suppressed hepatic triglyceride and VLDL synthesis. Inulin and FOS are short- and long- chain, linear fructans, consisting of fructose monomers bound with b-2,1-glycoside bonds [149]. Fructans are not viscous and incapable to bind with bile acids in the intestine. Thus, the hypolipeamic effects of FOS and inulin are attributed to SCFAs (mainly acetate, propionate, and butyrate) formed during the fermentation of fructans in the large intestine, which may inhibit enzymes involved in triglyceride and cholesterol synthesis in the liver in humans and rats [150,151]. It has been proved that dietary inulin-type fructans have reducing effects on TG and cholesterol in rodent studies. In human, most studies support positive effects of fructans in lipid metabolism, but the conflicting results still exist, especially in healthy subjects [151]. In addition, the consumption of iS such as soybean oligosaccharides and chitooligosaccharides increased high-density lipoprotein (HDL)-cholesterol in rats [152] and chicken [153] studies. A decreased serum low-density lipoprotein (LDL)-cholesterol with increased serum HDL-cholesterol was reported in Sprague-Dawley hypercholesterolemic-induced rats fed inulin [154]. A similar result was observed whereby serum total lipids, cholesterol, and LDL-C and VLDL-C were decreased whereas HDL-C was increased with 5% MOS supplementation in hyperlipidemic albino rats [155]. High-density lipoprotein cholesterol is involved in the reverse cholesterol transport, scavenging and removing bad cholesterol from the body. Although the mechanism of increasing effect of iS on HDL-cholesterol is unclear, it can be another explanation for hypolipeamic effects of iS activity.

## 4. Conclusions

This review primarily covers gut function-enhancing properties of iS and their metabolic effects in rodents and rabbits. The majority of the studies reviewed herein suggest prebiotic effects of the ingestion of iS, which include improvement of the microenvironment in the hindgut and constipation, as well as ameloriation in the nutrient metabolism, especially mineral absorption in the hindgut. Future studies are needed to evaluate long-term impact of iS supplementation as low caloric food supplement on mineral bioavailability. In particular, more clinical results are necessary in special cases such as patients suffering from mineral malabsorption or bone mineral losses due to aging, obesity or diabetes. In addition, more long-term designed studies are warranted to establish the effects of a prolonged supplementation with iS. Due to their beneficial effects on enhancing N utilization and micronutrient absorption, the use of iS offers great potentials not only in improving gut health and animal production, but also in decreasing environmental pollution via less excretion of N and minerals from animals.
